# A girl with a giant fibrolipoma in her thoracic cavity: a rare case report

**DOI:** 10.1186/s13256-019-2032-9

**Published:** 2019-05-07

**Authors:** Gang Wang, Chun Wu, Yi Wang, Jiangtao Dai

**Affiliations:** 1 Department of Cardio-Thoracic Surgery, Children’s Hospital, Chongqing Medical University, Chongqing, People’s Republic of China; 2 Pediatric Intensive Care Unit, Children’s Hospital, Chongqing Medical University, Chongqing, China; 3 Ministry of Education Key Laboratory of Child Development and Disorders, China International Science and Technology Cooperation Base of Child Development and Critical Disorders, Chongqing Key laboratory of Pediatrics, Chongqing, 400014 China

**Keywords:** Fibrolipoma, Thoracic cavity, Case report

## Abstract

**Background:**

Fibrolipoma is an uncommon subtype of lipoma. On the basis of the results of a survey of the PubMed database, only approximately a dozen cases in children have been described in which the histological diagnosis of fibrolipoma has been confirmed. In children, fibrolipomas have been reported in the eyelids, ears, lips, nasopharynx, mouth, and other locations but not in the thoracic cavity. We present the case of a 15-year-old girl with a giant fibrolipoma in the left side of her thoracic cavity.

**Case presentation:**

A 15-year-old Chinese girl presented with chest tightness and exercise-induced shortness of breath of 4 months’ duration. Computed tomography revealed a giant mixed-density space-occupying lesion in the left side of her thoracic cavity, originating possibly from the pleura. Radiological findings were inconclusive and failed to exclude malignant mesenchymal tumor. After excluding malignant tumor with two needle biopsies and identifying the tumor’s feeding blood vessels by computed tomography angiography examination, our treatment plan was, first, embolization of the tumor’s blood vessels by digital subtraction angiography and, second, to remove the tumor by thoracotomy.

**Conclusions:**

Thoracic fibrolipoma in children is rare; its treatment is complete resection of the tumor. Pathological examination of the removed tissue is the gold standard for diagnosis. Early computed tomography and magnetic resonance imaging examinations are helpful to determine the extent and nature of the tumor and to reduce damage of the surrounding organs. Preoperative examination of tumor markers, ultrasound-guided biopsy, and preoperative digital subtraction angiography tumor vascular embolization are important preoperative preparations to ensure surgical resection.

## Introduction

Fibrolipoma is an uncommon subtype of lipoma [1]; it is characterized by the presence of adipose tissue and abundant amounts of fibrous tissues in the tumor that are completely separated from surrounding tissues [1–3]. On the basis of the results of a survey of the PubMed database [4–12], only approximately a dozen cases in children have been reported in which the histological diagnosis of fibrolipoma has been confirmed. In children, fibrolipomas have been reported in the eyelids, ears, lips, nasopharynx, mouth, esophagus, peritoneum, and other locations but not in the thoracic cavity. To aid clinicians in further understanding the clinical manifestations, diagnosis, and treatment of children with thoracic fibrolipoma, we present the case of a 15-year-old girl with a giant fibrolipoma in the left side of her thoracic cavity.

## Case presentation

**Fig. 1:**
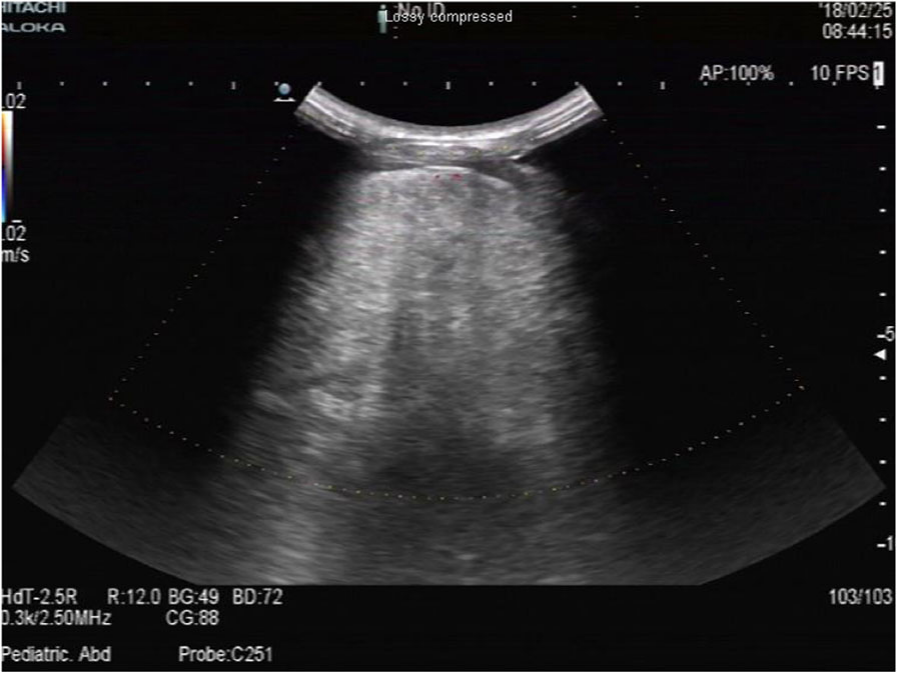
Giant substantial echo of the chest

**Fig. 2:**
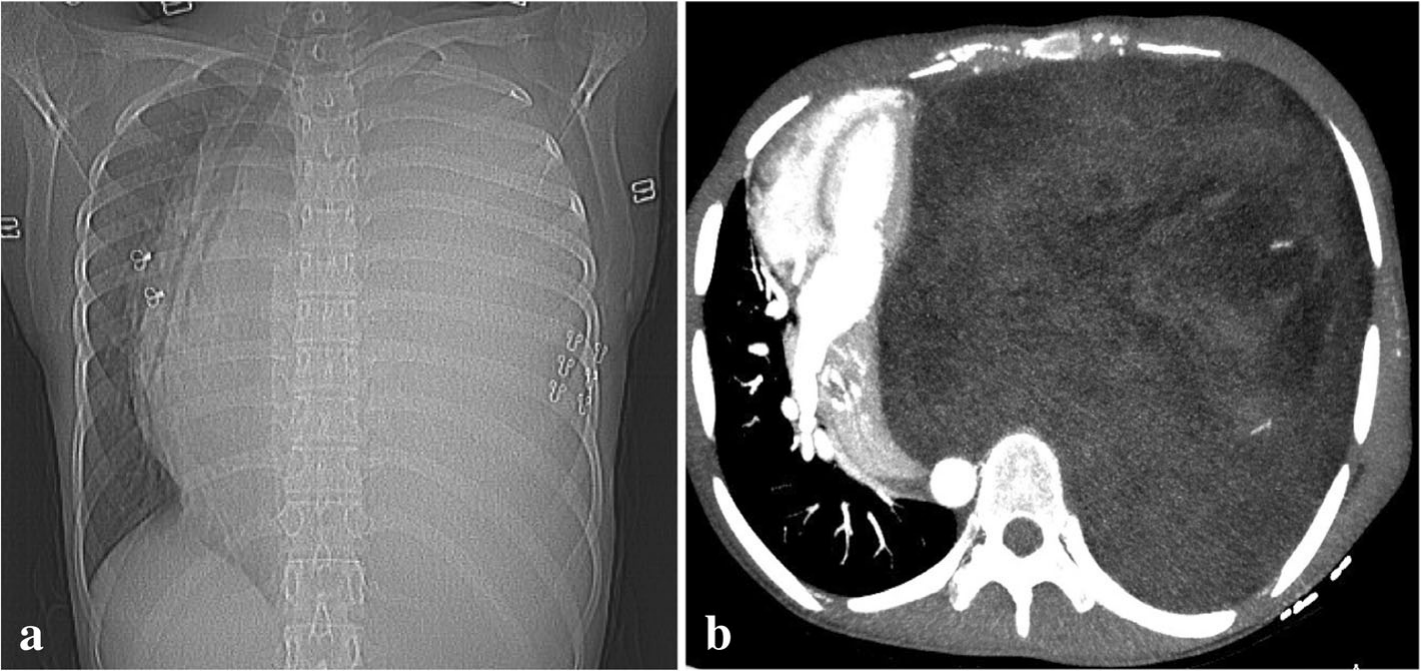
Computed tomography angiography images (**a** is sagittal image and **b** is cross-sectional image), giant mixed density masses, substantial compression of the left lung and heart

A 15-year-old Chinese girl, a rural middle school student, presented with chest tightness and exercise-induced shortness of breath of 4 months’ duration. During this time, she had no obvious explanation for the gradual development of exercise-induced shortness of breath, with occasional pain in her left shoulder and back, chest tightness, limited activity, occasional cough, no fever, no hot flashes, no night sweats, and no weight loss. She was able to climb only two to three flights of stairs before resting. In the past 4 months she had not sought treatment because of her heavy learning load and insufficient attention from her parents. She was brought to our hospital by her parents for treatment because the chest tightness and the shortness of breath became more severe after activity. A physical examination on admission revealed body temperature of 36.5 °C pulse of 95 beats/minute, and blood pressure of 105/60 mmHg; there were no abnormalities in a neurological examination. A medical examination by a specialist revealed no cyanosis or dyspnea at rest. Her left thorax was full with flatness to percussion. There was breath sound asymmetry, with absence of breath sounds on the left and no dry or wet rales. The apex of her heart beat was located in the midline of her right clavicle. Blood analysis revealed white blood cells (WBC) of 5.15 × 10^9^/L, platelets (PLT) of 118 × 10^9^/L, red blood cells (RBC) of 5.15 × 10^12^/L, and hemoglobin (HB) of 144 g/L; liver and kidney function were normal. Tumor markers were as follows: alpha fetoprotein (AFP) 0.46 ng/ml, human chorionic gonadotropin (hCG) < 1 mIU/ml, ferritin 152 ng/ml, neuron-specific enolase (NSE) 12.4 ng/ml, urinary vanillylmandelic acid (VMA) 7.82 mg/24 hours. Chest color Doppler ultrasonography revealed a giant tumor with substantial echo and irregular shape. Color Doppler flow imaging (CDFI) revealed substantial visible star-like blood flow signals in the left side of her thoracic cavity (Fig. 1). Enhanced computed tomography (CT) revealed a giant mixed-density mass in the left side of her thoracic cavity, part of which was fat density, measuring 323.6 mm × 193.1 mm × 175.6 mm. The enhancement of the parenchymal part of the tumor was not obvious, but it was slightly enhanced after a delay. Tumor vessels were seen in the arterial phase. Her left lung was substantially compressed into dense shadows. Her mediastinum, heart, and thoracic aorta were substantially compressed and pushed to the right. Her right lung was also compressed without hilar lymph node enlargement. The left eighth posterior intercostal artery was enlarged and the branches of the vessels entered the tumor. The branching vessels of the celiac trunk also sent enlarged and twisted branches into the tumor. Airway reconstruction revealed that her trachea and bronchus were compressed, the left part of the bronchus and the distal end were not seen, and her left diaphragm was depressed. A CT scan revealed a giant mixed-density space-occupying lesion in the left side of her thoracic cavity, originating possibly from the pleura. Radiological findings were unable to excluded malignant mesenchymal tumor (Fig. 2a, b).

**Fig. 3:**
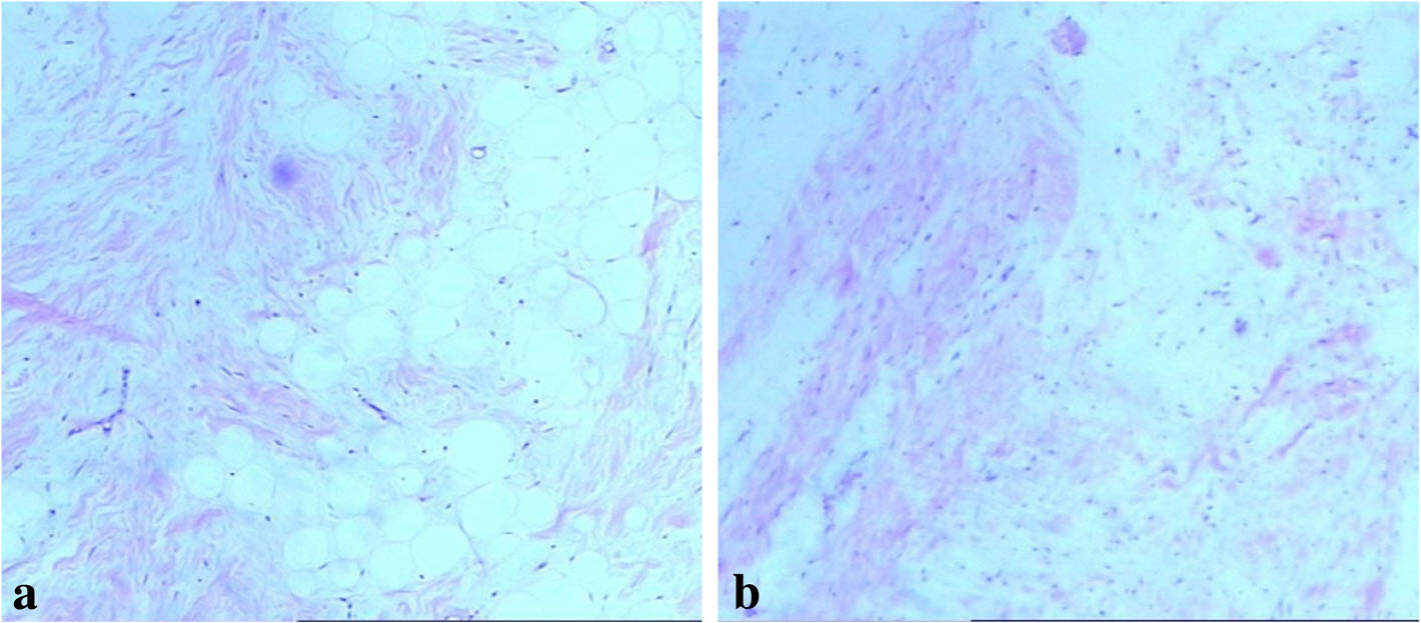
**a** First pathological examination; **b** second pathological examination. Both microphotograph showing mature fibrous adipose tissue, striated muscle tissue and rich mesodermal tissue (hematoxylin and eosin, × 100)

Because of the giant tumor and the inability to exclude malignant mesenchymal tumor on radiological findings, a fine-needle aspiration biopsy was performed under ultrasound guidance to clarify the nature of the tumor. Pathological examination revealed mature fibrous adipose tissue and striated muscle tissue (Fig. 3a). However, the pathologists were suspicious of the result in relation to the CT scan and suggested that the tumor be re-examined; therefore, an ultrasound-guided percutaneous needle biopsy was performed again. The second time, the pathological diagnosis was possible teratoma with mature fibrous adipose tissue, striated muscle tissue, and rich mesodermal tissue (Fig. 3b).

**Fig. 4:**
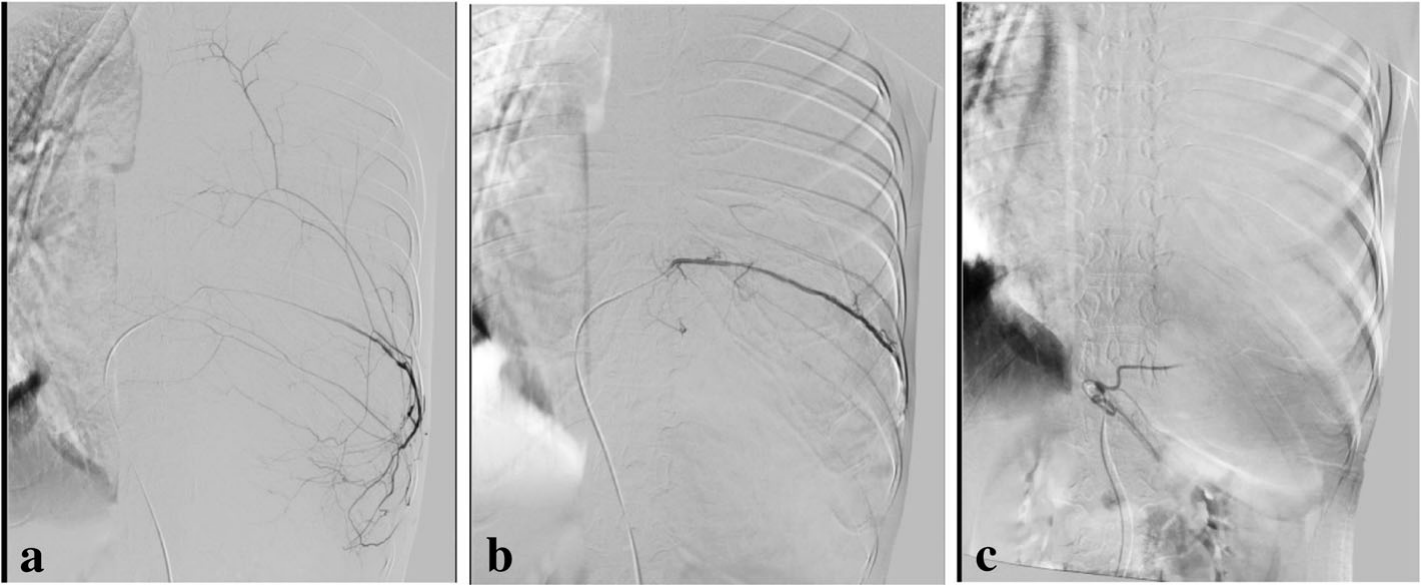
**a** Approximately 70% of the tumor was supplied by an enlarged branch of the target artery at the left eighth intercostal space. **b** Approximately 20% of the tumor was supplied by the branch of the target artery in the celiac trunk. **c** Embolize the two target arteries

After excluding malignant tumor with two needle biopsies and identifying the tumor’s feeding blood vessels by CT angiography (CTA) examination, our treatment plan was, first, embolization of the tumor’s blood vessels by digital subtraction angiography (DSA) and then, second, to remove the tumor by thoracotomy. DSA revealed that approximately 70% of the tumor was supplied by an enlarged branch of the target artery at the left eighth intercostal space, and approximately 20% of the tumor was supplied by the branch of the target artery in the celiac trunk (Fig. 4a, b). Using a 0.035 inch × 150 cm guidewire and a 2.7 F microcatheter, a 560 μm gelatin sponge+contrast medium 15 ml suspension was injected through the microcatheter to achieve embolization of the two target arteries. After DSA embolization, the result was satisfactory (Fig. 4c).

**Fig. 5:**
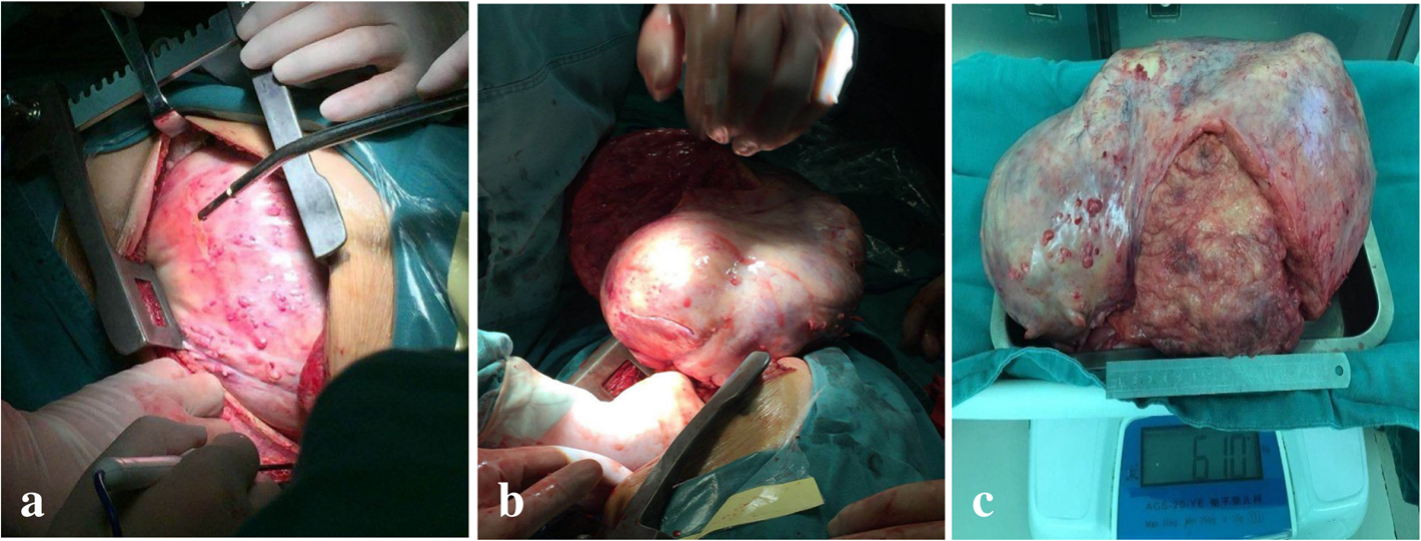
**a** Tumor shape in the thoracic; **b** tumor shape after removal of thoracic cavity; **c** tumor weighing

She had a mild febrile reaction after embolization. On the third day after embolization, she underwent thoracotomy through the left fifth costal oblique incision. The lesion occupied the entire left side of her thoracic cavity; the tumor had a solid intact capsule and moderate texture (Fig. 5a). Her left lung was severely compressed, her left hemidiaphragm was depressed, and her mediastinum, heart, and thoracic aorta were displaced to the right. After the tumor was partly removed from the center, the remaining tumor was removed from the left side of her thoracic cavity by being folded up and down. To avoid the occurrence of recurrent pulmonary edema, her left lung was suppressed by hand for approximately 15 minutes after removing the tumor and the lung was slowly re-expanded (Fig. 5b). The lesion size was approximately 360 mm × 200 mm × 180 mm, and the tumor weight was approximately 6.1 kg (Fig. 5c).

**Fig. 6:**
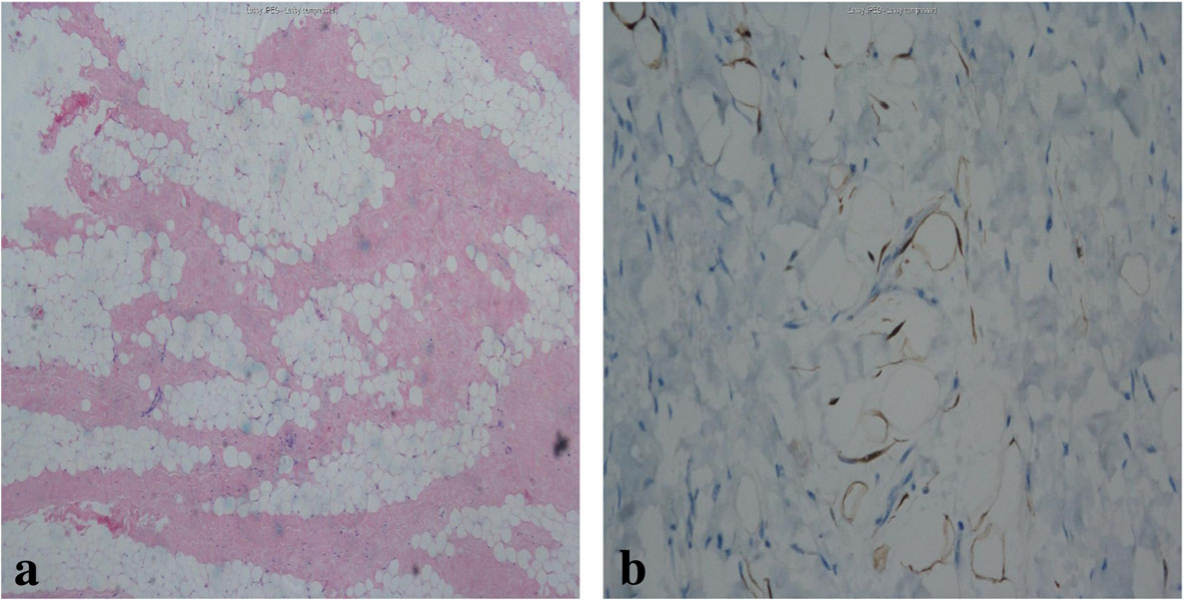
**a** Microphotograph showing adipose tissue hyperplasia in the wide fiber interval (hematoxylin and eosin, × 40) **b** SMA blood vessels (+), (immunohistochemistry image × 100)

Postoperative pathological results were as follows: there was adipose tissue hyperplasia in the wide fiber interval; immunohistochemistry revealed melanA (−), desmin (−), Ki67 individual cells (+), CD34 fibers and blood vessels (+), bcl-2 (−), s100 (+), and smooth muscle actin (SMA) blood vessels (+) (Fig. 6a, b). The pathological diagnosis was fibrolipoma.

**Fig. 7:**
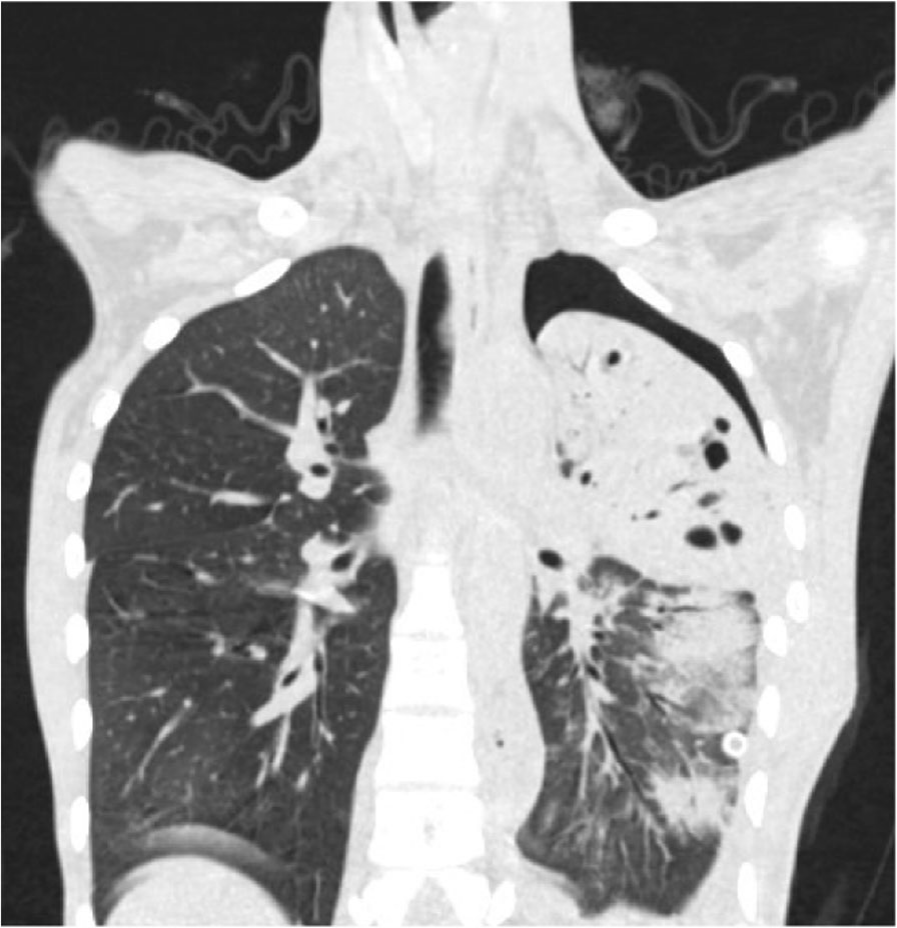
Computed tomography image 1 week after operation: substantial consolidation and several cystic lesions of various sizes

**Fig. 8:**
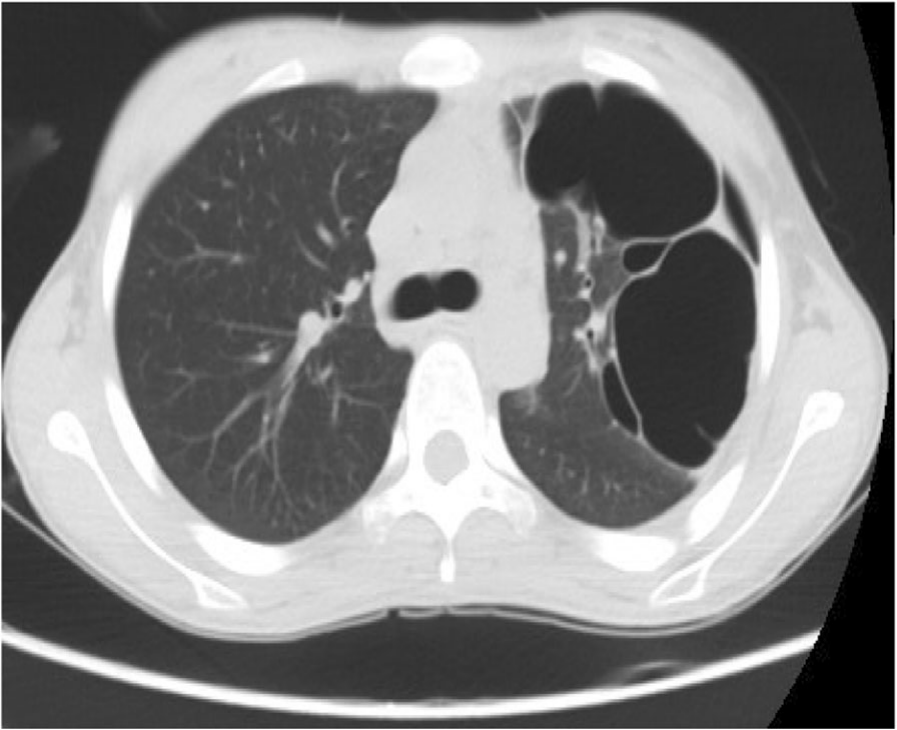
Computed tomography image 1 month after surgery: consolidation improved and cystic lesions did not decrease

**Fig. 9:**
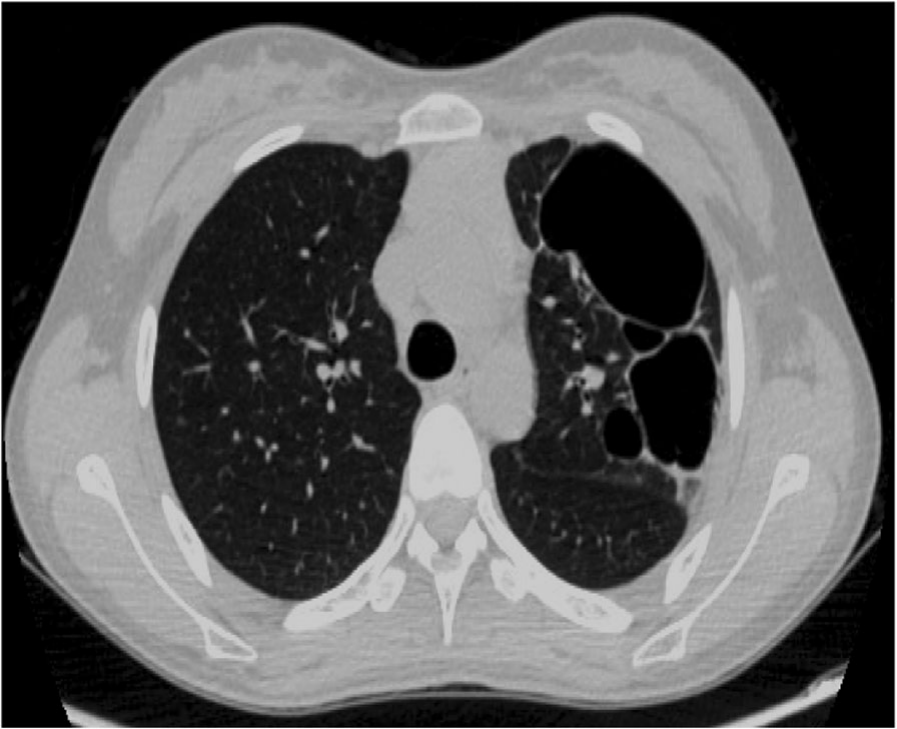
Computed tomography image 6 months after operation: cystic lesions did not improve

CT results 1 week after surgery revealed no tumor in our patient’s thoracic cavity. The upper lobe of her left lung was substantially consolidated with several cystic lesions of various sizes (Fig. 7). After two fibrobronchoscopy treatments, CT results 1 month after surgery revealed improved atelectasis with several persistent cystic lesions in the left lung (Fig. 8). CT results 6 months after surgery revealed that the several cystic lesions of various sizes in her left lung did not improve compared with the previous result (Fig. 9**)**. She was recommended to follow up for 1 or 2 years. If there were still cystic lesions in her left lung, her pulmonary cystic lesions would be removed.

Therefore, for giant thoracic tumors, if malignant tumor cannot be excluded before surgery and it is thought that they pose a high risk of bleeding during the operation, we recommend ultrasound-guided biopsy first, then DSA tumor vascular embolization, and finally surgical resection. The girl’s huge thoracic tumor was safely and completely removed by this method, and the prognosis is good. Otherwise, it may lead to unresectable tumors and the risk of massive hemorrhage.

## Discussion

Thoracic tumors of children are common in neurogenic, tracheal, and esophageal tumors of the posterior mediastinum as well as in lymphoma, leukemia, thymoma, and teratoma of the anterior mediastinum. Pulmonary blastoma, myofibroblastoma, pleural mesothelioma, liposarcoma, and others are rare. It is essential to know the nature and size of thoracic tumors because the treatments will be quite different. Lipoma is a benign tumor composed of mature adipocytes. If there are many fibrous tissues in the lipoma, it is called a fibrolipoma. These characteristically grow by simple expansion in a well-encapsulated fashion without the tissue infiltration that is more characteristic of liposarcomas [13]. Because fibrous tissues and adipose tissues are tumor parenchyma and the texture is slightly tough [2, 14, 15], they are classified as variants of conventional lipoma by the World Health Organization (WHO). Reported cases of fibrolipoma in recent years show that they can invade several parts of the body, including the face, lips, throat, trachea, esophagus, spermatic cord, abdominal cavity, and other locations [4–16]; however, there have been no reports of occurrence in children’s thoracic cavity. The clinical features of fibrolipomas vary according to their rate of growth, size, and location. On clinical examination, a fibrolipoma usually presents as an asymptomatic and slowly growing mass [1, 14]; therefore, it is difficult to value the incidence of the tumor. There is no definite etiology of fibrolipoma. Some authors believe that it is congenital, caused by endocrine disorders, or that it is a product of degenerative fibrous tumors, or is caused by the maturation of lipoblastoma [16]; its treatment is complete surgical resection. In the present case, our patient had no systemic disease or any history of trauma initially, as well as no family history of fibrolipoma, but she sought medical help when it appeared as a symptom and a functional problem. Because of its giant size, the case should be differentiated from lipoma, pulmonary blastoma, pleural mesothelioma, thoracic myofibroblastoma, and liposarcoma, especially from the latter that can be divided into well-differentiated and poorly differentiated liposarcoma. Well-differentiated liposarcoma contains many fat components and has clear borders and regular morphology. Sometimes, it is difficult to differentiate; however, necrotic cystic change or calcification in the tumor is helpful for making the differential diagnosis. It is easy to differentiate poorly differentiated and highly malignant liposarcoma with irregular morphology and infiltration into surrounding tissues containing a small amount of, or even no, fatty tissue. Ultrasound-guided biopsy is a reliable method of identification; however, the case was also misdiagnosed as teratoma.

It is a problem for surgeons to determine how to safely and effectively treat giant thoracic tumors of children. In this case, the first ultrasound-guided needle biopsy was performed to exclude malignant tumors, then embolization of the tumor vessels was accomplished by DSA to reduce intraoperative bleeding, and, finally, the whole tumor was removed. Although the tumor was giant and the visual field was poor, the tumor could be resected from a part to the whole because of a complete capsule and the reduction of the tumor’s blood supply after embolization by DSA. In particular, the heart, large vessels, esophagus, and trachea should be protected during surgery. Furthermore, another important treatment step is that the surgeon needs to suppress the affected lung by hand for about a dozen minutes and loosen slowly to avoid re-expansion pulmonary edema after resection of the giant thoracic tumor. Due to insufficient attention from parents and children, long-term compression of giant tumors results in pulmonary cystic lesions. If the cystic lesions do not improve after 1 or 2 years, it may be possible to remove them by reoperation.

## Conclusion

Thoracic fibrolipoma in children is rare; its treatment is complete resection of the tumor. Pathological examination of the removed tissue is the gold standard for diagnosis. Early CT and MRI examinations are helpful to determine the extent and nature of the tumor and to reduce damage to the surrounding organs. Preoperative examination of tumor markers, ultrasound-guided biopsy, and preoperative DSA tumor vascular embolization are important preoperative preparations to ensure surgical resection. The protection of the affected lung during surgery is also a problem that surgeons should not ignore.

## Abbreviations

AFP: Alpha fetoprotein
CDFI: Color Doppler flow imaging
CT: Computed tomography
CTA: Computed tomography angiography
DSA: Digital subtraction angiography
HB: Hemoglobin
hCG: Human chorionic gonadotropin
NSE: Neuron-specific enolase
PLT: Platelets
RBC: Red blood cells
SMA: Smooth muscle actin
VMA: Vanillylmandelic acid
WBC: White blood cells
WHO: World Health Organization
